# Characterization of the Immune Response during Infection Caused by *Clostridioides difficile*

**DOI:** 10.3390/microorganisms7100435

**Published:** 2019-10-10

**Authors:** Zohar Hamo, Maya Azrad, Orna Nitzan, Avi Peretz

**Affiliations:** 1 The Azrieli Faculty of Medicine, Bar Ilan University, Safed 13100, Israel; ZHamo@poria.health.gov.il (Z.H.); ONitzan@poria.health.gov.il (O.N.); 2 The Clinical Microbiology Laboratory, Baruch Padeh Medical Center, Poriya 15208, Israel; MAzrad@poria.health.gov.il; 3 Unit of Infectious Diseases, Baruch Padeh Medical Center, Poriya 15208, Israel

**Keywords:** immune response, *Clostridioides difficile*, disease severity, biomarker, cytokines

## Abstract

The high risk of complications and death following *Clostridioides difficile* infection (CDI) requires identifying patients with severe disease and treating them accordingly. We characterized the immune response of CDI patients in relation to infection severity. Concentrations of 28 cytokines and chemokines were measured in serum samples, obtained from 54 CDI patients within a median timeframe of 24–48 h after laboratory confirmation of *C. difficile* infection. Demographic and clinical data were retrospectively collected from medical records. Disease severity score was determined by “Score indices for *Clostridioides difficile* infection severity”. Of 54 patients (mean age, 76.6 years, 61.1% female), 38 (70.4%) had mild disease and 16 (29.6%) had moderate disease. Seven cytokines were associated with a more severe CDI: granulocyte-macrophage colony-stimulating factor (*p* = 0.0106), interleukin (IL)-1β (*p* = 0.004), IL-8 (*p* = 0.0098), IL-12p70 (*p* = 0.0118), interferon-α (*p* = 0.0282), IL-15 (*p* = 0.0015), and IL-2 (*p* = 0.0031). Additionally, there was an increased T-helper 1 response in more severe cases of CDI. Cytokines may serve as biomarkers for early prediction of CDI severity. Better and earlier assessment of illness severity will contribute to the adjustment of medical treatment, including monitoring and follow-up.

## 1. Introduction

*Clostridioides difficile* infection (CDI) is one of the leading causes of nosocomial diarrhea. The infection often occurs following antibiotic treatment that alters the gut microbiota, enabling *C. difficile* to thrive and cause symptoms ranging from mild diarrhea to severe colitis and death [1].

Several factors contribute to CDI pathogenesis including the bacteria’s abilities to produce toxins and to form spores. The observation that *C. difficile* may colonize healthy people without causing disease has advocated the idea that the host immune response also contributes to CDI progress, among other host-dependent factors [2].

The gastrointestinal system is the first line of defense against bacteria, viruses, fungi, and parasites. During bacterial infections, the innate immune system is activated, primarily in the intestinal mucus. The toxins released by *C. difficile* in the intestine cause dissociation of tight junctions and loss of epithelial integrity. In order to impede the damage and to prevent toxins from spreading into the bloodstream, T-helper cells (Th cells) release cytokines and activate a cascade of pro-inflammatory cytokines and other mediators that propagate the inflammatory process [2]. Naive Th cells differentiate into Th1, Th2, or Th17 effector cells that are antagonistic to each other and secrete different cytokines. Additionally, each pathogen elicits a typical Th response. It was found that two principal cytokines, interleukin (IL)-6 and tumor necrosis factor alpha (TNFα), significantly exacerbate inflammation caused by *C. difficile* in accordance with infection severity [3].

The emergence of a particularly virulent *C. difficile* strain called Ribotype 027 (NAP1) over the last decade has caused a significant increase in CDI frequency, illness severity and mortality. NAP1 is characterized by a relatively high resistance to fluoroquinolone antibiotics, higher sporulation rate and increased secretion of toxins than other strains, as well as secretion of binary toxin [4]. This toxin disaggregates the actin cytoskeleton and induces effusion in intestinal epithelial cell cultures, eventually leading to apoptosis [5]. Several NAP1 outbreaks were identified in hospitals and in long-term care facilities in Israel [4]. 

At present, there is no reliable marker for the assessment of CDI severity and prognosis. Recently, cytoskeletal Tropomyosin (Tpm) was suggested as a new marker for CDI [6]. Although the sensitivity of Tpm detection in feces was high (93.2%), its specificity was quite low (53.7%). Another publication has proposed the detection of volatile organic compounds (VOC) in patients’ feces [7]. However, this requires thermal desorption-gas chromatography-mass spectrometry or the development of other instrumentation. In light of CDI’s high risk of complications, early and more accurate evaluation of its severity would be extremely valuable for rapid and specific treatment administration. Therefore, it is important to find a specific biomarker that could indicate disease severity. Consequently, it may be possible to adjust the treatment and follow-up required for each CDI patient by measuring these markers. In this study, we characterized the immune response of CDI patients in relation to infection severity. We hypothesized that a more severe disease is associated with the release of higher levels of cytokines and chemokines and hence, with a stronger immune response.

## 2. Materials and Methods

### 2.1. Study Population

The study population included patients diagnosed with CDI that were hospitalized at the Baruch Padeh Medical Center, between November 2015 and May 2017. Patients with sepsis due to causes other than CDI, and bacteremia were excluded from the analysis. The study was approved by the Poriya Baruch Padeh Medical Center Ethics Committee (Approval number, POR-0085-15, 08/02/2016). All of the participants signed an informed consent prior to enrolling in the study.

All CDI cases were confirmed for toxigenic *C. difficile* by stool examination using the GeneXpert *C. difficile* polymerase chain reaction (PCR) assay (Cepheid, Sunnyvale, CA, USA), identifying three targets: Toxin B, Binary Toxin, and presence of tcdC deletion. Laboratory parameters such as: C-reactive protein, white blood cell levels, as well as the percentage of neutrophils and lymphocytes were taken from each patient’s medical record from the day the CDI was diagnosed.

### 2.2. Measurement of Cytokine Concentrations

Blood samples were collected within 24–48 h of the positive diagnostic result, and the serum was separated by centrifugation. Serum cytokine levels were determined using multiplex sandwich immunoassays with the Milliplex Human 41 Cytokine/Chemokine Premixed Kit (Millipore; Billerica, MA, USA) according to the manufacturer’s protocol. The kit includes 28 cytokines and chemokines: IL-1α, IL-1β, IL-1Ra, IL-2, IL-4, IL-5, IL-6, IL-7, IL-10, IL-12p40, IL-12p70, IL-13, IL-15, IL-17A, granulocyte-colony stimulating factor (G-CSF), granulocyte-macrophage colony-stimulating factor (GM-CSF), endothelial growth factor (EGF), eotaxin, fibroblast growth factor 2 (FGF2), IL-8, interferon gamma-induced protein 10 (IP-10), monocyte chemoattractant protein 1 (MCP-1), macrophage inflammatory protein α (MIP1α), macrophage inflammatory protein β (MIP1β), vascular endothelial growth factor (VEGF), tumor necrosis factor α (TNFα), interferon α2 (IFNα2) and interferon γ (IFNγ).

Serum of patients with positive toxigenic *C. difficile* results were combined with pre-mixed antibody-coupled magnetic fluorescent beads and assay buffer and incubated at 4 °C on a micro-titer plate shaker overnight. The following day, the samples were washed, pulled down using a magnetic plate washer and then incubated with biotin-conjugated secondary detection antibodies for one hour at room temperature. The samples were then washed again. A streptavidin-phycoerythrin detection solution was added to the samples and this mixture was incubated for 30 min at room temperature. Following incubation, sheath fluid was added to each sample well and the plate was read on the Luminex 200^TM^ florescence reader (Luminex Molecular Diagnostic; Toronto, ON, Canada). Results of samples that were below the kit’s identification threshold were excluded from the final analysis.

### 2.3. Analysis of T-Cell Responses

To understand the role of T-helper (Th) cells in CDI pathogenesis, Th1 and Th2 response was analyzed by calculating the ratio of IL-12p70 to IL-10, and the ratio of IL-12p70 to IL-5. The Th1 and Th17 response was analyzed by calculating the ratio of IL-12p70 to IL-17A, as previously described [8].

### 2.4. Disease Severity Scoring and Demographic Data Collection

CDI severity was evaluated by the severity score index (SSI) developed by Velazquez-Gomez et al. [9]. One point was given for each of the following parameters: changes in consciousness, pain/abdominal bulges, leukocytes above 20,000 cells/μL or below 1500 cells/μL, albumin below 2.5 mg/dL, ascites/colitis, tachycardia above 110 ppm, mean arterial pressure below 65 mmHg and transfer to the intensive care unit.

An SSI of 0–3 points was defined as mild CDI and an SSI of 4–7 points was defined as moderate CDI.

The following demographic and clinical data were retrospectively collected from the patients’ medical records: age, gender, community versus nosocomial-acquired CDI, death during hospitalization, white blood cell count, albumin level, creatinine level, and C-reactive protein level.

### 2.5. Toxin Detection

Toxins A and B were detected using the CerTest *Clostridium difficile* GDH+ Toxin A + B kit according to the manufacturer’s instructions (Certest Biotec, S.L, San Mateo de Gállego, Zaragoza, Spain). The kit is a colored chromatographic assay for qualitative detection of *C. difficile* antigen glutamate dehydrogenase and Toxins A and B in stool samples. The sample was mixed with a test solution that contains mouse monoclonal antibodies anti-GDH/Toxin A/Toxin B conjugated to red polystyrene latex. When GDH antigen/Toxin A/Toxin B is present in the sample, the antigen/toxin reacts with its specific antibodies in the test solution and this complex is captured by the antibodies in the test strips, resulting in a visible red line.

### 2.6. Statistical Analysis

The data were analyzed using SAS^®^ version 9.3 (SAS Institute, Cary, NC, USA). Categorical variables were summarized by number and percentage and continuous data were summarized by mean, standard deviation (SD), median, minimum and maximum. The Non-parametric Wilcoxon–Mann–Whitney Rank sum test for independent samples was applied for analyzing the differences in distribution of cytokine levels between mild and moderate CDI severity groups. 

All tests applied were two-tailed, and a *p* value of 5% or less was considered statistically significant. 

## 3. Results

### 3.1. Patient Epidemiologic Data

Fifty-four patients aged 46–98 years (mean age, 76.6 years, 61.1% female) positive for C. difficile were included in the study. Table 1 summarizes the demographic and clinical characteristics of the patients. Most CDI cases (37/54, 68.5%) were nosocomial, and the rest were community acquired. CDI severity was mild in 38 patients (70.4%) and moderate in 16 patients (29.6%). The most common isolated bacterial strains had both A + B toxin (34/54 patients, 63%). Six isolates (11.1%) had Toxin A and 14 (25.9%) had Toxin B.

In general, patients with CDI were characterized by higher values of white blood cells, albumin, creatinine, and C-reactive protein (compared with the normal range of these parameters) (Table 2).

### 3.2. Characterization of the Immune Response in CDI Patients

**Figure 1:**
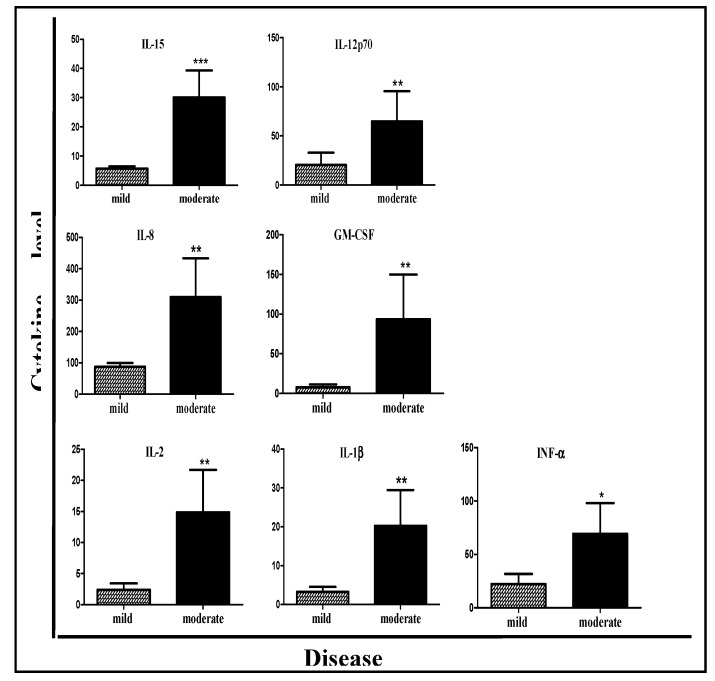
Serum inflammatory mediator levels correlate with disease severity of CDI patients. Moderate CDI patients had higher inflammatory mediator levels than patients with mild disease. * *p* < 0.05, ** *p* < 0.01, *** *p* < 0.001.

In order to find a biological marker that could correlate with disease severity, the level of 28 inflammatory mediators was measured in the patients’ serum and compared between patients with mild and moderate CDI (Table 3). Of these 28 inflammatory mediators, significantly higher levels of seven cytokines were found in the moderate CDI group when compared with the mild CDI group: GM-CSF (*p* = 0.0106), IL-1β (*p* = 0.004), IL-8 (*p* = 0.0098), IL-12p70 (*p* = 0.0118), INF-α (*p* = 0.0282), IL-15 (*p* = 0.0015), and IL-2 (*p* = 0.0031) (Table 3, Figure 1). In addition, there was a trend for higher release of IFN-γ in patients with moderate CDI (*p* = 0.07).

**Figure 2:**
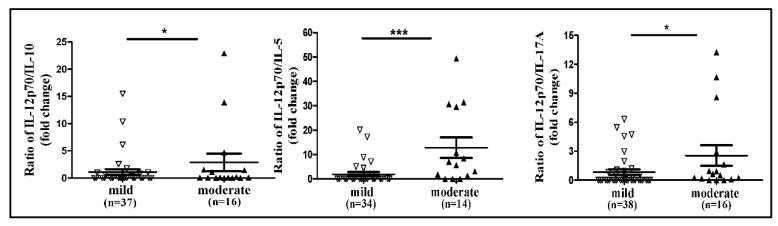
Ratio of Th1 to Th2 cells and Th1 to Th17 associated with severe CDI. *p < 0.05, ** *p* < 0.01, *** *p* < 0.001.

Assessment of the Th response showed that the IL-12p70/IL-5 ratio was 6.58-fold higher in the group of patients with moderate disease (mean 12.78) than in the group with mild disease (mean 1.94, *p* = 0.0001), suggesting an increased Th1 response in patients with a more severe disease (Figure 2).

The IL-12p70/IL-10 ratio was 2.61-fold higher in the group with moderate disease (mean 2.86) than in the group with mild disease (mean 1.095, *p* = 0.02). The IL-12p70/IL-17A ratio was 3.03-fold higher in the group with moderate disease (mean 2.55) than in the group with mild disease (mean 0.84, *p* = 0.023), suggesting a decrease in Th17 response in patients with more severe disease (Figure 2).

## 4. Discussion

In line with our hypothesis, we found an association between CDI severity and a stronger immune response. The levels of seven different cytokines were significantly higher in patients with moderate CDI: IL-15, IL-8, IL-2, IL-1β, INF-α, IL-12p70, and GM-CSF. These cytokines take part in the inflammatory response and are involved in its amplification, indicating their essential role in CDI pathogenesis.

IL-8 activates the chemotaxis mechanism for neutrophil recruitment and activation of innate lymphoid cells [10]. Previous studies have shown a positive correlation between CDI severity and high IL-8 levels in fecal samples of CDI patients [11,12]. High levels of fecal IL-8 also increase the risk of CDI recurrence [13]. Additionally, a specific polymorphism of IL-8 was associated with increased susceptibility to severe CDI [14]. Similar to IL-8, IL-1β is essential for host response and provides protection following infection and injury [15]. In addition to direct neutrophil recruitment, IL-1β also promotes IL-8 production, thus further increasing the levels of recruited neutrophils into the inflamed site [2]. Fecal concentration ratios of IL-1β/IL-Ra were significantly increased in patients with severe CDI compared to patients with mild CDI [11], supporting our findings.

GM-CSF promotes mucosal infections, including ones in the gastrointestinal tract [16,17]. It is involved in recruitment of neutrophils to the infection site [2], as was demonstrated in *C. difficile*-infected mice [18]. To the best of our knowledge, ours is the first report that shows that serum GM-CSF levels are associated with CDI severity. 

Neutrophil infiltration is a well-known feature of CDI and is believed to amplify the host immune response [19]. As IL-8, IL-1β and GM-CSF are all involved in the attraction of neutrophils to the inflammation center, it is reasonable that higher levels of IL-8, IL-1β and GM-CSF correlate with a more severe condition.

IL-2 and IL-15 were also significantly increased in patients with moderate CDI, as was previously reported [8]. IL-2 is secreted by lymphocytes and plays a central role in stimulating the proliferation of mucous lymphocytes, natural killer (NK) cells, and macrophages [20]. The main role of IL-2 is the expansion of Th cell populations through increased expression of high-affinity IL-2 receptors [20]. In addition, IL-2, together with IL-15, increase the number of NK cells [21,22,23,24] and promote NK cells to produce cytokines such as TNF-α, IFN-λ, and GM-CSF [25]. IL-15 indirectly affects neutrophil and monocyte chemotaxis to the infected site [26]. In macrophages, IL-15 promotes the production of pro-inflammatory cytokines such as TNF-α, IL-1, and IL-6 [27].

An additional virulence factor of *C. difficile* that affects the immune response is S-layer proteins (SLPs), which are recognized by the host’s Toll-like receptor 4 (TLR4). This interaction activates a cascade of events that eventually leads to the maturation of dendritic cells (DS), characterized by cytokine production [28]. It was shown that identification of pure *C. difficile* SLPs by TLR4 activates the Nf-kB signaling pathway, and consequently, leads to Th1 and Th17 responses [29]. Th1 cells induce the inflammatory responses that are part of the host cellular immunity, while Th17 is more involved in tissue destruction [30]. In our current study, IL-12p70 levels were significantly increased in patients with moderate CDI. As this cytokine is produced by Th1 and Th17 cells, this result suggests a considerable involvement of Th1 and Th17 in severe CDI. IFN-α also induces Th1 response, as was demonstrated by the significantly higher levels of IFN-α noted in patients with moderate CDI [31]. IFN-α increases the recruitment of cytotoxic T-cells, NK cells, and phagocytes. In addition, IFN-α promotes the differentiation of naive T-cells into Th1 phenotype [32,33]. This is the first time that IFN-α has been found to be associated with the severity of CDI. 

We found an increase in release of IFN-γ in patients with moderate CDI. IFN-γ is a potent mediator of innate immunity at mucosal sites, including the gastrointestinal tract [34,35,36]. Neutrophils produce IFN-γ in response to CDIs [36]. Abt et al. [37] has suggested a critical role for IFN-γ-producing type I innate lymphoid cells in mediating host survival during *C. difficile* colitis.

For further elucidation of Th1, Th2, and Th17 involvement in severe CDI, we calculated the ratio of IL-12p70/IL-10, and IL-12p70/IL-5 as a measure for Th1/Th2 response proportion, and the IL-12p70/IL-17A as a measure for Th1/Th17 response. We found a higher Th1/Th2 ratio in moderate disease compared with mild disease. These results match the change in the levels of specific Th1 cytokines, such as IL-2, in the mild and moderate CDI groups. Although a recent study of 36 CDI patients, has found opposite results, according to which there was a shift from Th1 to Th2 response when the disease was more severe [8], it is possible that blood sampling was performed at a different stage of the disease than our sampling. It is well-established that immune tolerance against commensal bacteria leads to the induction of protective T regulatory cells (iTreg) in the gut, while antibiotic treatment, which changes the microbiome, down-regulates iTreg and up-regulates Th1 and Th17 cells, resulting in a mild pro-inflammatory response. As *C. difficile* infection further damages the epithelium and increases cellular immune response, such a condition may result in a more intense inflammatory reaction [3]. Th2 cells, which regulate humoral immunity, may be involved in a later stage of CDI [38]. Regarding Th17 response, we found that the IL-12p70/IL-17A ratio was higher in moderate disease compared with mild disease, indicating that Th17 response did not increase when the disease was more severe. This finding does not correlate with previous reports in which Th17 response increased with disease severity [8,39] and was correlated with higher odds for death post-infection [39]. One reason for this discrepancy may be that we investigated the disease at an early stage, in which Th17 response had not yet increased, or that patient co-morbidities caused variance in the types of cytokine secretion and T-cell response.

We expected to find increased levels of IL-6 and TNF-α levels in more severe CDI, because both of these pro-inflammatory cytokines have been shown to increase during the activation of the innate immune system. Additionally, a previous publication has shown that IL-6 levels increased with disease severity [39]. However, no statistically significant differences were noted in the level of these cytokines between mild and moderate CDI. A possible explanation is that the patients’ co-morbidities masked part of the immune response. The study has several limitations. First, it is limited by its small sample size. Second, CDI severity was measured by one of several scales available, and only mild and moderate CDI were correlated with cytokine levels. Third, although serum sampling was done within 24–48 h of receiving a positive result for *C. difficile* presence, the samples may not have been collected at the same stage in terms of the immune response. Therefore, for further studies, we suggest collecting several blood samples on sequential days during hospitalization in order to detect the time point at which a change in cytokine levels occurs. Fourth, no comparison was made to intestinal disease not caused by *C. difficile*.

In summary, our results point to the involvement of several pro-inflammatory cytokines in CDI pathogenesis, which is reflected in recruitment and activation of neutrophils, NK cells, and T-cell differentiation. In line with our hypothesis, disease severity correlated with the intensification of this immune response. In addition, the significant association between disease severity and increased levels of IL-15, IL-8, IL-2, IL-1β, INF-α, IL-12p70, and GM-CSF indicates that these cytokines may serve as effective diagnostic tools to predict disease severity in the case of moderate CDI. These cytokines are easy to measure and the results are obtained quickly. Therefore, we suggest that in parallel to the initial identification of CDI, testing cytokine levels in the blood as part of routine blood tests may be effective for predicting disease severity, in addition to other clinical data. Recently, another inflammatory marker—Procalcitonin, a precursor peptide of calcitonin—has been found to be elevated during CDI and may be correlated with disease severity. Measurement of this biomarker is easy and quick [40]. Better and earlier assessment of CDI severity will contribute to adjustment of the medical treatment, allowing for improved patient care and may reduce patient mortality. Further prospective studies are needed to investigate the efficacy of pro-inflammatory cytokines in the diagnosis and follow-up phases of CDI, as an alternative or as a supplement to blood tests.

## Figures and Tables

**Table 1:** Clinical and demographic characteristics of patients with *Clostridioides difficile* infection (CDI).

Parameter	N (%)
Gender	
Male	21 (38.9)
Female	33 (61.10)
Disease severity	
Mild	38 (70.4)
Moderate	16 (29.60)
In- hospital mortality	
Alive	39 (72.2)
Dead	15 (27.8)
Toxin production	
Toxin A	6 (11.1)
Toxin B	14 (25.9)
Toxins A + B	34 (63)
Infection acquisition	
Nosocomial	37 (68.5)
Community	17 (31.5)

**Table 2:** Patients’ biochemical parameters.

Biochemical Parameter	Mean ± SD	Median (min, max)	Normal Range
Albumin (g/dL)	2.6 ± 0.6	2.4 (1.1, 4.3)	3.4–5.4
Creatinine (mg/dL)	1.9 ± 1.5	1.2 (0.3, 7.8)	0.6–1.2
C-reactive protein (mg/L) *	71 ± 32.2	82.1 (6.4, 100)	0–5
Lymphocytes (%)	13.5 ± 11.2	9.4 (1.1, 60.7)	22–43
Neutrophils (%)	78.2 ± 13.3	83.1 (23.9, 96.2)	40–70
White blood cells (cells/µL)	15,803.3 ± 10,053.7	15,285 (1700, 70,000)	4300–10,800

* C-reactive protein above 100 mg/L was considered 100.

**Table 3:** Serum inflammatory mediators’ levels (cytokines, chemokines and growth factors) in patients with CDI divided into mild and moderate CDI groups.

Cytokine (pg/mL)		Mild (*n* = 38) Mean (Range)	Moderate (*n* = 16) Mean (Range)	*p*-Value
	Disease Severity			
EGF		95.7 (0–325.7)	122.2 (5.3–438.9)	0.8875
Eotaxin		156 (28.1–359.8)	140.5 (25.3–280)	0.7702
FGF2		66.6 (0–255.9)	215.8 (0–826.5)	0.0561
G-CSF		142.1 (0–1881.8)	172.4 (41.2–1009.2)	0.1463
GM-CSF		7.7 (0–140.3)	93.4 (0–877.4)	0.0106 *
IFN-γ		23.7 (0–149.4)	53.4 (0–319)	0.07
IL-10		25.4 (0–96.6)	122.5 (0–1116.4)	0.0924
IL-12p40		11.4 (0–149.9)	33.3 (0–344.2)	0.5672
IL-12p70		20.5 (0–466.2)	64.7 (0–447.8)	0.0118
IL-13		20.4 (0–507)	8.7 (0–100.8)	0.8731
IL-15		5.7 (0–16.6)	30.1 (0.7–131.9)	0.0015
IL-17A		23.4 (0–203.1)	30.2 (0–194.4)	0.3134
IL-1Rα		150 (0–925.5)	414.5 (0–2836.3)	0.132
IL-1α		45.6 (0–765)	115.4 (0–1330.1)	0.5601
IL-1β		3.2 (0–29.5)	20.3 (0–113.5)	0.004
IL-2		2.4 (0–36.2)	14.9 (0–104.7)	0.0031
IL-4		7.2 (0–209.4)	10.4 (0–83.4)	0.1327
IL-5		4.1 (0–33.9)	3.2 (0–26.6)	0.8765
IL-6		74 (0–657)	80 (0–296.5)	0.452
IL-7		9.2 (0–28.5)	14.4 (0–108.5)	0.8201
IL-8		88 (14.2–354.5)	309.8 (28.5–2033.5)	0.0098
INF-α		22.3 (0–297.4)	69.3 (0–399)	0.0282
IP-10		527.5 (0–2969.9)	640.1 (84–3806)	0.6873
MCP-1		945.3 (81.8–2817.7)	1090.9 (143.5–3659.2)	0.7682
MIP 1α		17.5 (0–56.1)	27 (0–85)	0.3619
MIP 1β		44.4 (5–190.3)	80.5 (13.9–295.6)	0.1404
TNFα		35.5 (15.2–89.6)	38.7 (9.7–89.7)	0.6854
VEGF		413.6 (41.9–1679.5)	1123.6 (0–9426)	0.7416

EGF, endothelial growth factor; FGF, fibroblast growth factor 2; G-CSF, granulocyte colony-stimulating factor; GM-CSF, granulocyte-macrophage colony-stimulating factor; IFN, interferon; IL, interleukin; IP-10, interferon gamma-induced protein; MCP-1, monocyte chemoattractant protein 1; MIP, macrophage inflammatory protein; VEGF, vascular endothelial growth factor. * Bold values symbolize statistical significance.
